# Brain perfusion and blood-brain barrier permeability in systemic lupus erythematosus patients: Associations with disease activity, cognitive dysfunction, fatigue and pain

**DOI:** 10.1016/j.ynirp.2024.100232

**Published:** 2024-12-31

**Authors:** Tim Salomonsson, Kristoffer A. Zervides, Andreas Jönsen, Malte Knutsson, Ronnie Wirestam, Jimmy Lätt, Anders A. Bengtsson, Linda Knutsson, Pia C. Sundgren

**Affiliations:** a Department of Clinical Sciences, Diagnostic Radiology, Lund University, Skåne University Hospital, 221 85, Lund, Sweden; b Department of Clinical Sciences, Rheumatology, Lund University, Skåne University Hospital, 222 42, Lund, Sweden; c Department of Medical Radiation Physics, Lund University, 221 85, Lund, Sweden; d Department of Medical Imaging and Physiology, Skåne University Hospital, 221 85, Lund, Sweden; e Department of Neurology, Johns Hopkins University School of Medicine, Baltimore, MD, 21287, United States; f F.M. Kirby Research Center for Functional Brain Imaging, Kennedy Krieger Institute, Baltimore, MD, 21205, United States; g Lund University Bioimaging Center, Lund University, 221 84, Lund, Sweden

**Keywords:** Systemic lupus erythematosus, Brain perfusion, Blood-brain barrier permeability, Dynamic susceptibility contrast MRI, Cognitive dysfunction, Fatigue, Pain

## Abstract

High disease activity, cognitive dysfunction (CD), fatigue and pain negatively affect the quality of life in patients with systemic lupus erythematosus (SLE). However, the impact on brain perfusion and blood-brain barrier (BBB) permeability remains incompletely understood. Therefore, we utilized 3 T dynamic susceptibility contrast magnetic resonance imaging to investigate these factors in a cohort of 66 female SLE patients. Normalized leakage corrected cerebral blood flow (CBF), cerebral blood volume (CBV), mean transit time (MTT), and the BBB leakage parameter K_2,_ were compared within the cohort by splitting the group into patients with and without each symptom respectively. Fourteen regions of interest were chosen, and the results were adjusted for age, disease duration, smoking and glucocorticoids. We found regional significant alterations in the different SLE subgroups compared to patients without each corresponding symptom, with patterns as follows: moderate to high disease activity (n = 17, decreased MTT, increased K_2_), CD in ≥1 domain (n = 36, decreased MTT, increased K_2_), CD in ≥2 domains (n = 20, increased CBF, CBV and K_2_), fatigue (n = 44, increased CBV and MTT), pain (n = 9, increased CBF and CBV, decreased MTT). Additionally, inverse correlations were found between cognitive scores and K_2_ in multiple areas, indicating increased BBB permeability with worse cognitive performance. To elucidate the underlying mechanisms, longitudinal studies should be conducted in a larger variation of patients, using different measurements of BBB disruption.

## Highlights

• Altered perfusion-based metrics in SLE patients with debilitating symptoms.
• Regional perfusion differences in patients with cognitive dysfunction.
• Increased blood-brain barrier leakage with worse cognitive performance.
• Widespread increase in brain perfusion in SLE patients with fibromyalgia.

## 1 Introduction

Systemic lupus erythematosus (SLE) is a chronic, autoimmune disease with a complex spectrum of manifestations, evident by the different criteria used in its classification (Petri et al., 2012). Depending on the definition, approximately half of all SLE patients present with nervous system manifestations attributed to the disease, known as neuropsychiatric SLE (NPSLE) (Unterman et al., 2011). The American College of Rheumatology (ACR) included 19 case definitions in the NPSLE nomenclature, ranging from focal central nervous system (CNS) syndromes such as cerebrovascular disease and seizures, to diffuse mood disorders and anxiety, and also peripheral nervous system syndromes including polyneuropathy and autonomic neuropathy (The American College of Rheumatology, 1999). Among the different neuropsychiatric manifestations, cognitive dysfunction (CD) is one of the most common with an estimated prevalence of up to 38 % of SLE patients (Rayes et al., 2018). CD is also one of the most debilitating manifestations, negatively impacting quality of life and participation in social life (Kim et al., 2021). Previous research suggests cognitive deficits commonly occur in domains of executive function, visual memory, attention, working memory and processing speed (Leslie and Crowe, 2018; Seet et al., 2021). Notably, SLE patients without overt NPSLE also display affected cognitive function more often than expected in the general population (Leslie and Crowe, 2018; Langensee et al., 2022). Several mechanisms could contribute to the pathogenesis of CD, including autoantibodies, proinflammatory cytokines, and complement factors leading to a disruption of the blood-brain barrier (BBB), and following antibody-mediated neurotoxicity as well as activation of complement and microglia (Seet et al., 2021). In addition to NPSLE manifestations, generally increased disease activity is also commonly associated with reduced quality of life (Shi et al., 2021). Similarly, other symptoms with a high prevalence have an equally debilitating impact in SLE, including fatigue (up to 90 %) and chronic pain (up to 30%) (Kim et al., 2021; Atzeni et al., 2011; Kawka et al., 2021). Collectively, these factors might represent general changes in the central nervous system as a result of acute and prolonged chronic inflammation, in early stages as well as in later stages of the disease (Kozora et al., 2006; Nichilatti et al., 2020). However, both fatigue and pain also occur in the absence of active disease, suggesting multifactorial causes, including organ damage and comorbidities, evident by the overlap between the symptoms and with conditions such as primary fibromyalgia (Atzeni et al., 2011; Kawka et al., 2021; Nichilatti et al., 2020).

Utilizing perfusion imaging in SLE has previously yielded inconclusive results, both when studying patients with NPSLE, increased disease activity or specific symptoms (Azizi et al., 2024). In SLE patients with active disease, findings have ranged between generally reduced brain perfusion to focally increased perfusion in areas such as the cingulate gyrus, suggesting regional involvement (López et al., 2003; Zhuo et al., 2020; Wang et al., 2012). Diverse findings related to CD in SLE have also been published, with some studies suggesting no differences (Olazarán et al., 2009; Waterloo et al., 2001), and others showing inconclusive results from widespread perfusion deficits to focal decreases or increases (Driver et al., 2008; Oh et al., 2011; Wang et al., 2023; Liu et al., 2024a). To study potential BBB disruption, dynamic susceptibility contrast (DSC) magnetic resonance imaging (MRI) has shown increased leakage in white matter (WM) hyperintensities in SLE, possibly indicating inflammatory processes with increased BBB permeability in active disease (Salomonsson et al., 2023). Additionally, dynamic contrast enhanced (DCE) MRI has revealed a global increase in BBB permeability, correlating with CD in SLE (Kamintsky et al., 2020; Hanly et al., 2022). The BBB may also be affected when developing symptoms such as chronic pain and fatigue, although this has not yet, to the best of our knowledge, been studied with perfusion MRI in SLE (Kawka et al., 2021; Nichilatti et al., 2020; Omdal et al., 2005).

Based on these previous findings, we decided to investigate the hypothesis that SLE patients with debilitating symptoms exhibit altered patterns of brain perfusion and increased BBB permeability. To our knowledge, DSC-MRI has not been used to study these parameters extensively in different subgroups of SLE patients and might therefore further contribute to the understanding of the underlying mechanisms. Thus, this study was conducted with the aim of evaluating cerebral perfusion and BBB leakage in relation to disease activity, CD, fatigue and pain in a cohort of SLE patients.

## 2 Materials and methods

### 2.1 Participants and disease metrics

This study was conducted in an established study group of 66 SLE patients from the tertiary rheumatology outpatient clinic at Skåne University Hospital, Lund, Sweden. The patients were consecutively recruited independently of symptomatology or disease activity, and fulfilled at least four of the Systemic Lupus International Collaborating Clinics (SLICC) criteria for SLE, including at least one clinical and one immunological manifestation (Petri et al., 2012). Inclusion criteria were female gender, age between 18 and 55 years and right-handedness to reduce inter-group differences in brain perfusion due to biological gender and handedness or age-related MRI abnormalities. An additional inclusion criterion was fluency in the Swedish language, in order to be able to perform the neurocognitive testing. Exclusion criteria were contraindications to undergoing MRI, including claustrophobia, pacemaker or other metal implants, pregnancy and contrast agent allergy. Within the context of this study, no healthy controls were included as the perfusion sequence requires administration of gadolinium-based contrast agent, which may accumulate in the brain across multiple scans (Kanda et al., 2015). Disease activity was assessed according to the SLE Disease Activity Index 2000 (SLEDAI-2K), with scores <4 defined as indicative of low activity, and scores ≥4 indicative of moderate to high disease activity (Gladman et al., 2002). Organ damage was assessed according to the SLICC/ACR Damage Index (SDI) (Gladman et al., 1996). The patients were evaluated according to the Fatigue Severity Scale (FSS), with fatigue defined as a score ≥36, in accordance with the definition of clinically significant fatigue in previous studies (Krupp et al., 1989). Chronic pain was diagnosed in accordance with the ACR 1990 classification of fibromyalgia (Wolfe et al., 1990). Visual analogue scales (VAS) 100 mm for fatigue and pain were also reported, asking the patients to assess the severity of the symptoms during the last two weeks.

### 2.2 Neurocognitive testing

One patient did not participate in cognitive testing and was excluded from this part of the analysis. Individual neurocognitive testing was performed by a neuropsychologist using the computerized test battery CNS Vital Signs (CNS-VS), as previously described (Langensee et al., 2022; Gualtieri and Johnson, 2006). The battery consists of seven established tests, which, in total, covers twelve different domains: verbal memory, visual memory, motor speed, processing speed, executive function, simple attention, reaction time, composite memory, psychomotor speed, cognitive flexibility, complex attention, and the neurocognition index (NCI) which reflects the global performance (Gualtieri and Johnson, 2006). The results were normalized to an age-matched healthy population to calculate standard scores, and individual test results were excluded according to the Validity Indicator algorithm in the software (Gualtieri and Johnson, 2006). CD was defined as a standard score ≤79 (≤−1.4 SD), corresponding with the cut-off for moderate impairment in CNS-VS (Gualtieri and Johnson, 2006). Patient groups were split based on CD in any domain (any domain deficit, ADD), in at least two domains (multiple domain deficit, MDD) and normal cognition (NC), in accordance with previously suggested response criteria in SLE (Mikdashi et al., 2007).

### 2.3 Magnetic resonance imaging

All participants underwent neuroimaging using a 3 T MRI (MAGNETOM Skyra, Siemens, Erlangen, Germany), including T1-and T2-weighted sequences, and DSC-MRI perfusion imaging with post-processing in the nordicICE software tool (NordicNeuroLab, Bergen, Norway), as described in detail in a previous study (Salomonsson et al., 2023). The perfusion analysis generated values of the cerebral blood flow (CBF), cerebral blood volume (CBV) and mean transit time (MTT), where MTT = CBV/CBF according to the central volume theorem. The perfusion parameters were normalized to the corresponding values in normal appearing WM in the bilateral middle cerebellar peduncles. This region of normalization was chosen in order to maintain consistency with previous methodology used in SLE patients, to improve reproducibility (Azizi et al., 2024; Salomonsson et al., 2023; Papadaki et al., 2019). The values were also corrected for potential extravasation of the contrast agent using a linear fitting algorithm, which returned the leakage parameter K_2_, reflecting the degree of extravascular leakage due to BBB dysfunction (Boxerman et al., 2006; Quarles et al., 2009). Since K_2_ can show both positive and negative values, originating from a shortening of T1 and T2∗, respectively, the estimates were converted to absolute values (i.e. only positive values) to better represent and compare the fraction of leakage (Quarles et al., 2009).

**Fig. 1:**
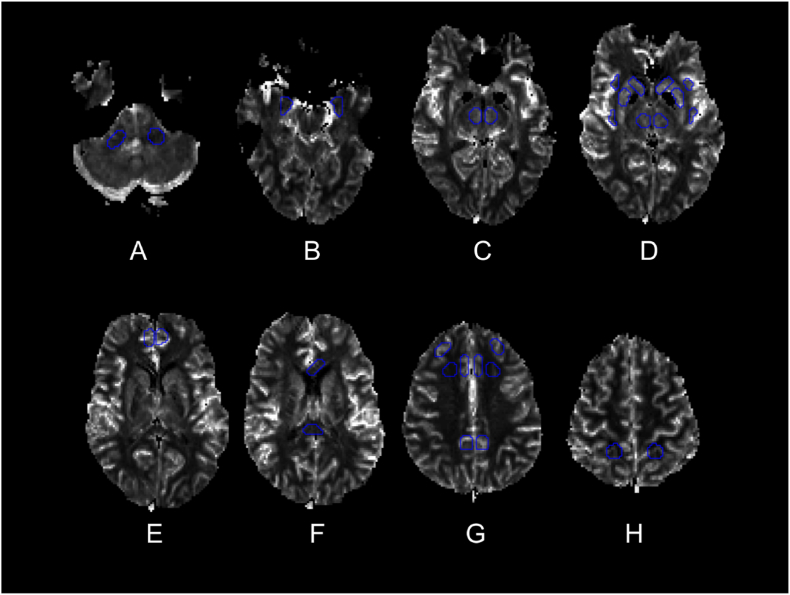
Placement of regions of interest on images of cerebral blood flow in a systemic lupus erythematosus patient. A. Middle cerebellar peduncles for normalization. B. Medial temporal lobe. C. Hypothalamus. D. Nucleus caudatus, putamen, thalamus, anterior and posterior insula. E. Ventromedial prefrontal cortex. F. Anterior and posterior corpus callosum. G. Dorsolateral prefrontal cortex, frontal white matter, anterior and posterior cingulate cortex. H. Superior parietal lobule.

Maps of CBF, CBV, MTT and K_2_ were analyzed using the in-house software Evaluation-Graphic User Interface (EvalGUI), developed by Markus Nilsson and colleagues at Lund University, Lund, Sweden. Regions of interest (ROIs) were placed in the CBF maps, with guidance of structural T1-and T2-weighted images, bilaterally in 14 predefined areas of the brain (Fig. 1): medial temporal lobe (MTL), hypothalami, nuclei caudatus, putamina, thalami, frontal WM, superior parietal lobules (SPL), dorsolateral (DL) and ventromedial (VM) prefrontal cortices (PFC), as well as both anterior and posterior ROIs in the insulae, corpus callosum and cingulate cortices. Each ROI was manually drawn in EvalGUI and measured 40 voxels in size, except for the ROIs in the insulae which were 20 voxels each. Any visible alterations in the MRI sequences including high values in singular CBF voxels (indicating intravascular space), null voxels or structural abnormalities such as WM hyperintensities were avoided.

### 2.4 Statistical analyses

All statistical analyses were performed using IBM SPSS version 29.0 (IBM, New York, NY, USA). The entire SLE group was split and compared regarding perfusion and leakage parameters (CBF, CBV, MTT, K_2_) using analysis of covariance, adjusting for age, disease duration, smoking and glucocorticoid medication, with post hoc Tukey HSD. The following parameters were used separately for each analysis: moderate to high disease activity (SLEDAI-2K score ≥4 vs ≤ 3), clinically significant fatigue (FSS ≥36 vs ≤ 35), pain (fibromyalgia diagnosis yes vs no), and CD according to the CNS-VS scores (NC vs CD in ≥1 and ≥ 2 domains respectively). Partial correlation was also performed, adjusting for age, disease duration, smoking and glucocorticoids, between the perfusion-based values and the following: SLEDAI-2K, standard scores in each cognitive domain, FSS, VAS fatigue and VAS pain. For comparison of categorical variables, the Chi-square test was performed. The statistical significance level was set to α = 0.05, and a correlation coefficient ≥0.4 or ≤ −0.4 indicated at least moderately strong correlation. Weak correlations were not reported due to assumed clinical non-significance. The study was considered exploratory, therefore no corrections for multiple comparisons were made.

## 3 Results

### 3.1 Patient characteristics

In total, 66 SLE patients were included for analysis (mean age at the time of the MRI scan 36 years, SD 9 years, range 18–52 years), presented in Table 1. In general, disease activity and disease damage were low, and the median disease duration was 10 years. More than half of the patients presented with CD in at least one domain, and the results on CNS-VS are presented in Supplementary Table 1. Two-thirds had clinically significant fatigue, and 9 patients had a diagnosis of chronic pain, according to the fibromyalgia classification. Ongoing medications at the time of the study included glucocorticoids (79 %), hydroxychloroquine (79 %), other disease-modifying antirheumatic drugs (DMARDs) (59 %), and antihypertensives (30 %). Current or history of smoking was noted in 23 patients.

**Table 1:** Clinical characteristics, ongoing medications and sizes of the different groups used to split the 66 SLE patients included in the analysis.

Clinical characteristics	
Age [years], mean (SD)	36 (9)
Disease duration [years], median (range)	10 (0–26)
SLICC SLE criteria, total [n], median (range)	7 (3–13)
SLICC SLE criteria, clinical [n], median (range)	4 (2–8)
SLICC SLE criteria, immunological [n], median (range)	3 (1–5)
SLEDAI-2K, median (range)	2 (0–18)
SDI, median (range)	0 (0–5)
FSS, median (range)	45 (0–63)
VAS fatigue, median (range)	61 (1–100)
VAS pain, median (range)	20 (1–99)
Smoking, n (%)	23 (35 %)
Glucocorticoid treatment, n (%)	52 (79 %)
Glucocorticoid treatment, daily dose [mg], median (range)	5 (0–25)
Hydroxychloroquine treatment, n (%)	52 (79 %)
Other DMARD treatment except hydroxychloroquine, n (%)	39 (59 %)
Antihypertensive treatment, n (%)	20 (30 %)
**Groups in the MRI analyses**	
Moderate to high disease activity, n (%)	17 (26 %)
CD in ≥1 domain, n (%)	36 (55 %)
CD in ≥2 domains, n (%)	20 (30 %)
Fatigue, n (%)	44 (68 %)
Fibromyalgia, n (%)	9 (14 %)
SLE: Systemic lupus erythematosus. SD: Standard deviation. SLICC: Systemic Lupus International Collaborating Clinics. n: number. SLEDAI-2K: Systemic Lupus Erythematosus Disease Activity Index 2000. SDI: SLICC/American College of Rheumatology Damage Index. FSS: Fatigue Severity Scale. VAS: Visual analogue scale. DMARD: Disease-modifying antirheumatic drug. CD: Cognitive deficit.	

### 3.2 Disease activity and MRI data

Patients with moderate to high disease activity showed decreased MTT in the right MTL and right nucleus caudatus, as well as increased K_2_ in the posterior corpus callosum, compared to patients with low disease activity, summarized in Table 2. There were no significant differences in disease duration, SDI score or smoking between the groups. The group with moderate to high disease activity was significantly older (mean age 38 vs 32 years, p = 0.03), had a higher daily dose of glucocorticoids (mean dose 7.5 vs 4.1 mg, p = 0.045) and were more commonly prescribed other DMARDs besides hydroxychloroquine (82 vs 51 %, p = 0.02), and antihypertensive treatment (53 vs 22 %, p = 0.02), compared to the group with low disease activity. No moderately strong correlations were found between SLEDAI-2K scores and perfusion or permeability estimates (data not shown).

**Table 2:** Normalized perfusion-based values (CBF, CBV and MTT) and absolute values of the leakage parameter K_2_, in the regions of interest that displayed significant differences between SLE patients with each specific symptom and the corresponding control group of SLE patients.

Region of interest	Symptom	Without symptom	P value
**Moderate to high disease activity**			
R MTL, MTT (norm.)	0.86	0.96	0.002
R Nc. caudatus, MTT (norm.)	0.78	0.83	0.047
Post. corpus callosum, K_2_ (min^−1^)	0.52	0.32	0.011
**CD in ≥1 domain**			
R Thalamus, MTT (norm.)	0.89	0.95	0.016
L Post. cingulate cortex, K_2_ (min^−1^)	0.22	0.15	0.046
**CD in ≥2 domains**			
L Frontal WM, CBF (norm.)	0.93	0.81	0.021
L Frontal WM, CBV (norm.)	0.96	0.85	0.034
Ant. corpus callosum, CBF (norm.	1.46	1.24	0.029
Ant. corpus callosum, CBV (norm.)	1.40	1.16	0.014
L Putamen, K_2_ (min^−1^)	0.41	0.31	0.039
L Post. cingulate cortex, K_2_ (min^−1^)	0.27	0.16	0.004
**Fatigue**			
L Hypothalamus, CBV (norm.)	2.09	1.84	0.044
R MTL, MTT (norm.)	0.96	0.89	0.025
SLE: Systemic lupus erythematosus. norm.: normalized. L: Left. R: Right. Nc: Nucleus. Ant: Anterior. Post: Posterior. WM: White matter. MTL: Medial temporal lobe. PFC: Prefrontal cortex. VM: Ventromedial. SPL: Superior parietal lobule. CBF: Cerebral blood flow. CBV: Cerebral blood volume. MTT: Mean transit time. K_2_: Blood-brain barrier leakage parameter. CD: Cognitive dysfunction.			

### 3.3 Cognitive dysfunction and MRI data

The significant differences between patients with and without CD are summarized in Table 2. Patients with CD in ≥1 domain showed significantly decreased MTT in the right thalamus and increased K_2_ in the left posterior cingulate cortex. Patients with CD in ≥2 domains showed significantly increased CBF and CBV in the left frontal WM and anterior corpus callosum, and increased K_2_ in the left putamen and left posterior cingulate cortex. There were no significant differences in age, disease duration, SLEDAI-2K or SDI score between the groups, nor in occurrence of smoking or medications, for any of the comparisons.

**Fig. 2:**
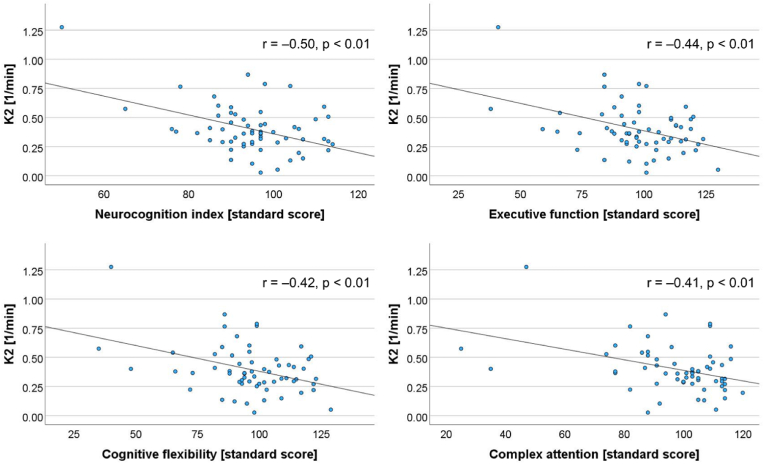
Representative examples of significant and at least moderately strong correlations between the blood-brain barrier leakage parameter K_2_ and cognitive performance, here shown in the right putamen and correlated with standard scores for executive function, cognitive flexibility, complex attention and the neurocognition index in the whole SLE group.

Significant and at least moderately strong negative correlations were found between worse cognitive function in specific domains and absolute values of K_2_ in multiple areas of the brain, shown in Supplementary Table 2, with representative examples in Fig. 2. The NCI, executive function, cognitive flexibility and complex attention correlated with K_2_ in the right putamen, and motor speed with K_2_ in the right posterior insula. Simple attention correlated with K_2_ in the left MTL, left anterior insula, left anterior cingulate cortex, right SPL, as well as bilaterally in the thalami, dorsolateral PFC and posterior cingulate cortices. The average K_2_ across all ROIs correlated with scores in reaction time and simple attention. There was also a significant negative correlation between MTT in the right hypothalamus and standard scores in motor speed (r = −0.4, p = 0.01).

### 3.4 Fatigue and MRI data

Patients with fatigue showed significantly increased CBV in the left hypothalamus and MTT in the right MTL, compared to patients without fatigue, summarized in Table 2. There were no significant differences in age, disease duration, SLEDAI-2K or SDI score between the groups, nor in occurrence of smoking or medications. No moderately strong correlations were found between FSS or VAS fatigue scores and perfusion or permeability measurements (data not shown).

### 3.5 Pain and MRI data

**Fig. 3:**
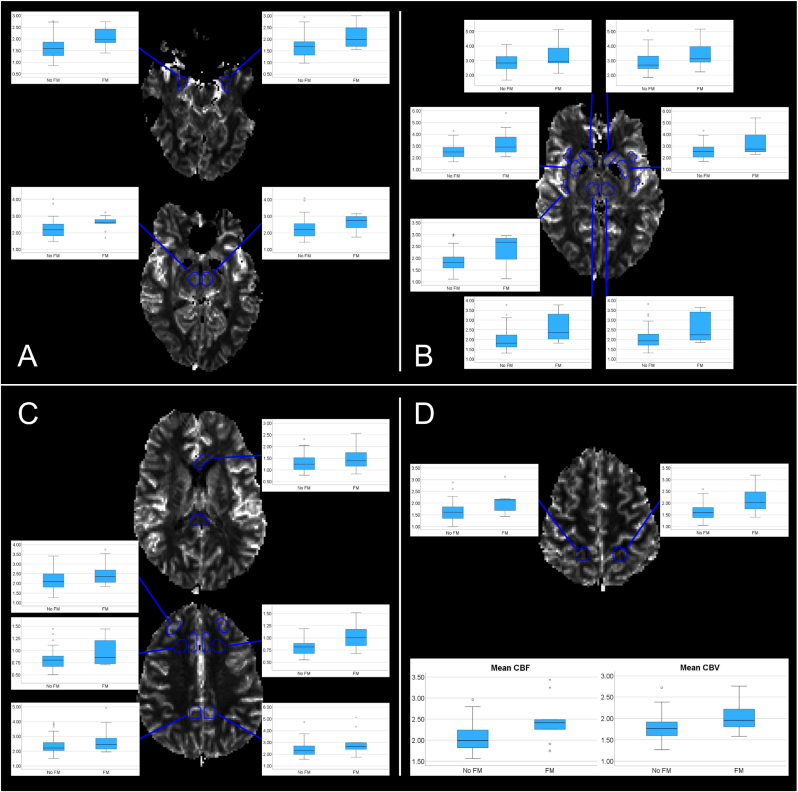
Representative regions of interest with boxplots showing the significant differences in cerebral blood flow (CBF) between patients without fibromyalgia (denoted no FM) and patients with fibromyalgia (denoted FM). A. Bilaterally in the medial temporal lobes and hypothalami. B. Bilaterally in the nuclei caudatus, putamina and thalami, as well as in the right posterior insula. C. Bilaterally in the posterior cingulate cortices, white matter in the frontal lobes, as well as in the right dorsolateral prefrontal cortex. D. Bilaterally in the superior parietal lobules, and boxplots showing the mean CBF and cerebral blood volume (CBV) across all regions.

SLE patients with fibromyalgia showed multiple increases in the perfusion-based metrics compared to SLE patients without fibromyalgia, summarized in Fig. 3 and Supplementary Table 3. The significant differences were seen in the anterior corpus callosum, right anterior and posterior insula, right DL PFC, and bilaterally in the MTL, hypothalami, caudate nuclei, putamina, thalami, posterior cingulate cortices, SPL and WM in the frontal lobes, as well as the average CBF and CBV across all ROIs. The fibromyalgia group also showed decreased MTT bilaterally in the thalami compared to the control group. There were no significant differences in age, SLEDAI-2K or SDI score between the groups, nor in occurrence of smoking or medications. The fibromyalgia group had significantly longer disease duration compared to the control group (median 20 vs 9 years, p = 0.03). No moderately strong correlations were found between VAS pain scores and perfusion or permeability measurements (data not shown).

## 4 Discussion

This cross-sectional study utilized DSC-MRI to evaluate cerebral perfusion and BBB permeability in a large cohort of female SLE patients. We wanted to study whether differences could be seen in patients with debilitating factors that each negatively impact the quality of life (Kim et al., 2021). Our results identify increased perfusion in frontal white matter areas and generally increased BBB permeability in patients with CD. We also found extensive patterns of increased perfusion in patients with fibromyalgia. These findings suggest alterations in the brain of SLE patients related to the different symptoms and will each be discussed separately in greater detail below.

In patients with moderate to high disease activity, there were few significant perfusion differences. This possibly reflects the clinically heterogeneous phenotypes of active SLE with increased variability of the metrics (Wang et al., 2012). However, we found increased K_2_ in the posterior part of the corpus callosum (splenium), indicating increased BBB permeability in the area. Notably, the splenium is located near the pineal gland and the subcommissural organ, both lacking a typical BBB. Therefore, inflammatory mediators may enter the cerebrospinal fluid (CSF) more easily from these areas. Additionally, as part of the circumventricular organs, they are thought to be involved in the recruitment of immune cells, thereby facilitating neuroinflammation (Schulz and Engelhardt, 2005). Some previous MRI studies have also seen perfusion and microstructural alterations in the corpus callosum as well as in the adjacent posterior cingulate gyrus, suggesting focal involvement of these regions in active disease (Zhuo et al., 2020; Wang et al., 2012; Julio et al., 2023).

A common chronic manifestation of SLE is CD (Rayes et al., 2018). However, the spectrum of CD may be quite diverse and complex to assess, as evidenced by the different test instruments used across studies and varying prevalence (Rayes et al., 2018). Using a clinician evaluation of CD in NPSLE may introduce classification bias. Therefore, we utilized CNS-VS which consists of commonly used standardized tests such as the symbol digit coding test and the Stroop test (Rayes et al., 2018; Gualtieri and Johnson, 2006). CNS-VS has also previously been used in SLE and validated in several patient cohorts (Rayes et al., 2018; Langensee et al., 2022; Gualtieri and Johnson, 2006; Nystedt et al., 2019). CD was investigated in patients with deficits in both ≥1 domain and ≥2 domains, to maintain reproducibility according to previously proposed criteria (Mikdashi et al., 2007). We found increased perfusion in white matter in the left frontal lobe and anterior corpus callosum, mainly in patients with MDD. This stands in contrast with some previous studies that found decreased perfusion in the frontal lobe with worse cognitive performance, which would have been indicative of ischemic lesions with vasculopathy as contributing factors to CD (Driver et al., 2008; Wang et al., 2023; Liu et al., 2024b). However, this may instead be interpreted alongside our other main finding, which showed multiple inverse correlations between K_2_ and cognitive scores in different domains. This indicates a general trend of increased BBB permeability with worse performance in patients with CD. Several regions showed this pattern, including the MTL, SPL, putamen, thalamus, insular, cingulate and prefrontal cortices. Combined, these results may indicate increased BBB permeability in different gray matter areas and compensatory hyperperfusion in frontal WM. Possible underlying mechanisms for our results may be related to alterations in the local microvasculature or increased neuronal demand, as previously seen with perfusion- and diffusion-weighted MRI sequences. Rates related to both capillary permeability, parenchymal diffusion, blood flow and extracellular free water have been found to be increased in SLE and correlated to cognitive performance (Gulati et al., 2017; DiFrancesco et al., 2020; Qian et al., 2022). This might indicate regional loss of the microvascular structure or degradation of the neurovascular unit, leading to a net increase in blood flow in patients with CD (Wiseman et al., 2016; DiFrancesco et al., 2020; Mayer et al., 2022). The compensatory mechanisms could also be attributed to hypermetabolism seen with positron emission tomography (PET) in the frontal lobe, basal ganglia and thalamus in SLE patients (Mackay et al., 2019). A previous study showed that these findings remained unchanged at later follow-up and were associated with CD as well as decreased microstructural integrity in adjacent WM tracts (Mackay et al., 2019). Indeed, this hypermetabolic response has also been found in murine models of SLE following autoantibody production with subsequent BBB disruption and worse cognitive performance (Vo et al., 2014). Both compensatory mechanisms initiated in the surviving neurons, activation of other local cells such as microglia, or possibly a combination of the two, may be responsible for these findings (Vo et al., 2014). The affected gray matter areas in our study population are also part of larger core resting state networks. Previous fMRI studies indicate that SLE patients might need increased hemodynamic responses particularly in frontoparietal regions, to maintain adequate cognition (Nystedt et al., 2019; Barraclough et al., 2015). Increased activation can also be seen when performing tasks related to working memory and executive function (Kozora et al., 2016). In general, increased perfusion could be related to neuroinflammation with vasodilation, increased BBB leakage and more acutely affected cognitive function. However, the patients with CD in our cohort did not display any significant signs of active disease compared to patients without CD, which raises the possibility of long-term compensatory mechanisms at play. Our findings also suggest in general that increased BBB permeability can be repeatedly detected with contrast-based MRI sequences in SLE patients with CD (Kamintsky et al., 2020; Hanly et al., 2022).

We also wanted to investigate whether SLE patients with pain or fatigue presented with any perfusion and permeability differences. We chose to mainly study patients with chronic pain according to the fibromyalgia classification and fatigue according to the FSS, which evaluates the impact of the patients' fatigue in daily life (Krupp et al., 1989; Wolfe et al., 1990). We also correlated the metrics with VAS assessing pain and fatigue during the last two weeks. However, this definition generated no associations that were at least moderately strong, possibly due to the subjectivity of these self-reported scales (Aitken, 1969). In patients with fibromyalgia, we found widespread patterns of increased perfusion in multiple areas of the brain. This is consistent with previous research showing brain activation and increased perfusion in both the thalamus, basal ganglia, SPL, frontal, prefrontal and insular cortices in patients with chronic pain (Wasan et al., 2011; Foerster et al., 2011; Shokouhi et al., 2016; Lee et al., 2019). It has also been suggested that persistent inflammation, tissue injury and nociceptive stimuli, might lead to biochemical, structural and functional changes in the brain, with alterations in the patient's perception of pain (Nichilatti et al., 2020). The results in our subgroup with established fibromyalgia further suggest perfusion alterations in pain processing regions as part of the changes associated with chronic pain. Conversely, in patients with fatigue, we instead found few perfusion differences, in line with an earlier SPECT study in SLE (Omdal et al., 2005). The only exceptions were decreased MTT in the right MTL and increased CBV in the left hypothalamus. Interestingly, the hypothalamus may be involved in the development of fatigue, possibly through mechanisms of neuroinflammation and hyperactivity in the hypothalamic-pituitary-adrenal axis (Trautmann, 2021). Therefore, this area warrants further investigations in SLE patients with fatigue.

Depending on the underlying characteristics of the tissue, the usage of K_2_ as a measurement of BBB permeability might lead to ambiguous results. The contrast agent may predominantly shorten either T1 or T2∗, corresponding to positive or negative K_2_ estimates, respectively (Quarles et al., 2009). For that reason, K_2_ was converted to absolute values to better reflect the degree of leakage. However, both effects can happen simultaneously, and a small value of K_2_ could either be a result of an intact BBB or be caused by simultaneous T1-and T2∗-relaxation effects, canceling each other out (Elschot et al., 2023). This might hamper the usage of K_2_ in detecting subtle permeability changes across larger regions, and other models of leakage correction may be used in future studies (Elschot et al., 2023). K_2_ could also be compared with other metrics, such as the CSF/plasma albumin ratio or the permeability-weighted K^trans^ obtained with DCE-MRI. Through such a comparison, effects related to neuroinflammation, damage to WM, the BBB or blood-CSF-barrier may then be further separated (Stock et al., 2017; Hillmer et al., 2023).

This study is not without limitations. As the design was exploratory, the results were not adjusted for multiple comparisons. Due to the limited number of studies on perfusion MRI in SLE, the data is heterogenous and scattered, as highlighted in a recent systematic review (Azizi et al., 2024). By choosing ROIs similar to the literature, we hope to shed light on possible perfusion and BBB alterations in other specific subgroups of SLE (Azizi et al., 2024). This exploratory approach reduces the likelihood of a type II error, suggesting regions that could be targeted to find truly important differences in further studies (Perneger, 1998). Moreover, this may guide future longitudinal research investigating whether these alterations remain consistent over time. Adequate adjustment for potential confounders is also necessary, and different factors should be considered depending on the subjects included. The patients in our study were in general well treated with a stable disease, making the cohort quite homogenous. In the analysis, we adjusted accordingly to reduce the influence of age, smoking, glucocorticoids and disease duration on a possible true difference. However, the patients were not assessed by a psychiatrist, and the potential influence of psychological factors such as depression or anxiety on the perfusion values, the performance on the cognitive tests, and reported perception of fatigue and pain, cannot be excluded (Kawka et al., 2021; Nichilatti et al., 2020; Papadaki et al., 2019; Giovacchini et al., 2010). Structural abnormalities such as cortical atrophy or volume loss in certain areas may also affect the cognitive results, as well as the perfusion-based values, and this could be considered in future research (Wang et al., 2023; Clement et al., 2018). The interpretation of general SLE-related effects is limited due to the inclusion of only female subjects and the exclusion of healthy controls (invoked by the potential adverse effects associated with gadolinium accumulation). Lastly, no definitive conclusions regarding causality can be made due to the cross-sectional design of the study.

## 5 Conclusion

We utilized DSC-MRI to identify different patterns of cerebral perfusion and BBB permeability in SLE patients with moderate to high disease activity, CD, fatigue and pain. In particular, we demonstrated areas with increased perfusion in patients with fibromyalgia and CD, and increased BBB leakage with worse performance on cognitive testing. Our findings suggest that perfusion MRI might further distinguish different clinical phenotypes of SLE, and reveal possible contributing mechanisms such as regional vasculopathy, microvascular alterations, compensatory responses, local neuronal hyperactivity or small vessel disease. Longitudinal research needs to be conducted in a larger variation of patients, adjusting for other covariates and using different measurements of BBB damage. Investigating the disease progression across follow-up studies may also further enable conclusions regarding any potential causality, prevention and reversibility of the different debilitating symptoms that are present in SLE.

## CRediT authorship contribution statement

**Tim Salomonsson:** Writing – original draft, Visualization, Software, Investigation, Formal analysis, Data curation. **Kristoffer A. Zervides:** Writing – review & editing, Methodology, Investigation, Data curation. **Andreas Jönsen:** Writing – review & editing, Resources, Methodology, Funding acquisition, Data curation, Conceptualization. **Malte Knutsson:** Investigation, Formal analysis, Data curation. **Ronnie Wirestam:** Writing – review & editing, Methodology, Funding acquisition. **Jimmy Lätt:** Writing – review & editing, Software, Resources, Data curation. **Anders A. Bengtsson:** Writing – review & editing, Resources, Project administration, Funding acquisition, Conceptualization. **Linda Knutsson:** Writing – review & editing, Resources, Methodology, Funding acquisition, Data curation, Conceptualization. **Pia C. Sundgren:** Writing – review & editing, Supervision, Resources, Project administration, Methodology, Funding acquisition, Conceptualization.

## Ethics statement

The project was approved by the relevant Swedish ethical authorities with permits 2012/254, 2012/677, 2014/778, and 2022-01960-02. All subjects signed written informed consent and could withdraw their consent at any time. Neither inclusion, exclusion nor findings from this study affected the management or treatment of the patients.

## Funding

This study was supported by funding from King Gustav V 80-years foundation (FAI-2019-0559), Skåne University Hospital Research Funding (2017–2024), Regional Research Support (Regionalt forskningsstöd 2019–2024, RegSkane-824561, RegSkane-2022-1171), Alfred Österlunds Research Foundation (2019–2022), the Anna-Greta Crafoord Foundation and the Greta and Johan Kock Foundation. The funding sources had no involvement regarding the study design, collection, analysis and interpretation of data, nor in the writing and submission of the article for publication.

## Declaration of competing interest

The authors declare that they have no known competing financial interests or personal relationships that could have appeared to influence the work reported in this paper.

## Data Availability

Data will be made available on request.

## Abbreviations

ACR: American College of Rheumatology
ADD: Any domain deficit
BBB: Blood-brain barrier
CBF: Cerebral blood flow
CBV: Cerebral blood volume
CD: Cognitive dysfunction
CNS: Central nervous system
CNS-VS: Central Nervous System Vital Signs
DCE: Dynamic contrast enhanced
DL: Dorsolateral
DMARD: Disease-modifying antirheumatic drug
DSC: Dynamic susceptibility contrast
fMRI: Functional magnetic resonance imaging
FSS: Fatigue Severity Scale
K_2_: Blood-brain barrier leakage parameter
MDD: Multiple domain deficit
MRI: Magnetic resonance imaging
MTL: Medial temporal lobe
MTT: Mean transit time
NC: Normal cognition
NCI: Neurocognition index
NPSLE: Neuropsychiatric systemic lupus erythematosus
PFC: Prefrontal cortex
ROI: Region of interest
SD: Standard deviation
SDI: Systemic Lupus International Collaborating Clinics/American College of Rheumatology Damage Index
SLE: Systemic lupus erythematosus
SLEDAI-2K: Systemic Lupus Erythematosus Disease Activity Index 2000
SLICC: Systemic Lupus International Collaborating Clinics
SPECT: Single-photon emission computed tomography
SPL: Superior parietal lobule
VAS: Visual analogue scale
VM: Ventromedial
WM: White matter

## References

[bib1] Aitken R.C. (1969). Measurement of feelings using visual analogue scales. Proc. Roy. Soc. Med..

[bib2] Atzeni F., Cazzola M., Benucci M., Di Franco M., Salaffi F., Sarzi-Puttini P. (2011). Chronic widespread pain in the spectrum of rheumatological diseases. Best Pract. Res. Clin. Rheumatol..

[bib3] Azizi N., Issaiy M., Jalali A.H., Kolahi S., Naghibi H., Zarei D. (2024). Perfusion-weighted MRI patterns in neuropsychiatric systemic lupus erythematosus: a systematic review and meta-analysis. Neuroradiology.

[bib4] Barraclough M., Elliott R., McKie S., Parker B., Bruce I.N. (2015). Cognitive dysfunction and functional magnetic resonance imaging in systemic lupus erythematosus. Lupus.

[bib5] Boxerman J.L., Schmainda K.M., Weisskoff R.M. (2006). Relative cerebral blood volume maps corrected for contrast agent extravasation significantly correlate with glioma tumor grade, whereas uncorrected maps do not. AJNR Am J Neuroradiol.

[bib6] Clement P., Mutsaerts H.-J., Václavů L., Ghariq E., Pizzini F.B., Smits M. (2018). Variability of physiological brain perfusion in healthy subjects - a systematic review of modifiers. Considerations for multi-center ASL studies. J. Cerebr. Blood Flow Metabol..

[bib7] DiFrancesco M.W., Lee G., Altaye M., Beebe D.W., Meyers-Eaton J., Brunner H.I. (2020). Cerebral microvascular and microstructural integrity is regionally altered in patients with systemic lupus erythematosus. Arthritis Res. Ther..

[bib8] Driver C.B., Wallace D.J., Lee J.C., Forbess C.J., Pourrabbani S., Minoshima S. (2008). Clinical validation of the watershed sign as a marker for neuropsychiatric systemic lupus erythematosus. Arthritis Rheum..

[bib9] Elschot E.P., Backes W.H., de Jong J.J.A., Drenthen G.S., Wong S.M., Staals J. (2023). Assessment of the clinical feasibility of detecting subtle blood-brain barrier leakage in cerebral small vessel disease using dynamic susceptibility contrast MRI. Magn. Reson. Imaging.

[bib10] Foerster B.R., Petrou M., Harris R.E., Barker P.B., Hoeffner E.G., Clauw D.J. (2011). Cerebral blood flow alterations in pain-processing regions of patients with fibromyalgia using perfusion MR imaging. AJNR Am J Neuroradiol.

[bib11] Giovacchini G., Mosca M., Manca G., Della Porta M., Neri C., Bombardieri S. (2010). Cerebral blood flow in depressed patients with systemic lupus erythematosus. J. Rheumatol..

[bib12] Gladman D., Ginzler E., Goldsmith C., Fortin P., Liang M., Urowitz M. (1996). The development and initial validation of the Systemic Lupus International Collaborating Clinics/American College of Rheumatology damage index for systemic lupus erythematosus. Arthritis Rheum..

[bib13] Gladman D.D., Ibañez D., Urowitz M.B. (2002). Systemic lupus erythematosus disease activity index 2000. J. Rheumatol..

[bib14] Gualtieri C.T., Johnson L.G. (2006). Reliability and validity of a computerized neurocognitive test battery, CNS Vital Signs. Arch. Clin. Neuropsychol..

[bib15] Gulati G., Jones J.T., Lee G., Altaye M., Beebe D.W., Meyers-Eaton J. (2017). Altered blood-brain barrier permeability in patients with systemic lupus erythematosus: a novel imaging approach. Arthritis Care Res..

[bib16] Hanly J.G., Legge A., Kamintsky L., Friedman A., Hashmi J.A., Beyea S.D. (2022). Role of autoantibodies and blood-brain barrier leakage in cognitive impairment in systemic lupus erythematosus. Lupus Sci Med.

[bib17] Hillmer L., Erhardt E.B., Caprihan A., Adair J.C., Knoefel J.E., Prestopnik J. (2023). Blood-brain barrier disruption measured by albumin index correlates with inflammatory fluid biomarkers. J. Cerebr. Blood Flow Metabol..

[bib18] Julio P.R., Caldeira T., Pinheiro G.R., Capello C.H., Fritolli R.B., Marini R. (2023). Microstructural changes in the corpus callosum in systemic lupus erythematous. Cells.

[bib19] Kamintsky L., Beyea S.D., Fisk J.D., Hashmi J.A., Omisade A., Calkin C. (2020). Blood-brain barrier leakage in systemic lupus erythematosus is associated with gray matter loss and cognitive impairment. Ann. Rheum. Dis..

[bib20] Kanda T., Fukusato T., Matsuda M., Toyoda K., Oba H., Kotoku J. (2015). Gadolinium-based contrast agent accumulates in the brain even in subjects without severe renal dysfunction: evaluation of autopsy brain specimens with inductively coupled plasma mass spectroscopy. Radiology.

[bib21] Kawka L., Schlencker A., Mertz P., Martin T., Arnaud L. (2021). Fatigue in systemic lupus erythematosus: an update on its impact, determinants and therapeutic management. J. Clin. Med..

[bib22] Kim M.Y., Sen D., Drummond R.R., Brandenburg M.C., Biesanz K.L., Kim A.H. (2021). Cognitive dysfunction among people with systemic lupus erythematosus is associated with reduced participation in daily life. Lupus.

[bib23] Kozora E., Ellison M.C., West S. (2006). Depression, fatigue, and pain in systemic lupus erythematosus (SLE): relationship to the American College of Rheumatology SLE neuropsychological battery. Arthritis Rheum..

[bib24] Kozora E., Uluğ A.M., Erkan D., Vo A., Filley C.M., Ramon G. (2016). Functional magnetic resonance imaging of working memory and executive dysfunction in systemic lupus erythematosus and antiphospholipid antibody-positive patients. Arthritis Care Res..

[bib25] Krupp L.B., LaRocca N.G., Muir-Nash J., Steinberg A.D. (1989). The fatigue severity scale. Application to patients with multiple sclerosis and systemic lupus erythematosus. Arch. Neurol..

[bib26] Langensee L., Mårtensson J., Jönsen A., Zervides K., Bengtsson A., Nystedt J. (2022). Cognitive performance in systemic lupus erythematosus patients: a cross-sectional and longitudinal study. BMC Rheumatol.

[bib27] Lee Y.C., Fine A., Protsenko E., Massarotti E., Edwards R.R., Mawla I. (2019). Brain correlates of continuous pain in rheumatoid arthritis as measured by pulsed arterial spin labeling. Arthritis Care Res..

[bib28] Leslie B., Crowe S.F. (2018). Cognitive functioning in systemic lupus erythematosus: a meta-analysis. Lupus.

[bib29] Liu H., Liu H., Tian B., Yang P., Fan G. (2024). Alterations in cerebral perfusion and corresponding brain functional networks in systemic lupus erythematosus with cognitive impairment. Res Sq.

[bib30] Liu H., Liu H., Tian B., Sun Z., Xiong W., Yang P. (2024). Alterations in cerebral perfusion and corresponding brain functional networks in NPSLE with cognitive impairment. Res Sq.

[bib31] López-Longo F.J., Carol N., Almoguera M.I., Olazarán J., Alonso-Farto J.C., Ortega A. (2003). Cerebral hypoperfusion detected by SPECT in patients with systemic lupus erythematosus is related to clinical activity and cumulative tissue damage. Lupus.

[bib32] Mackay M., Vo A., Tang C.C., Small M., Anderson E.W., Ploran E.J. (2019). Metabolic and microstructural alterations in the SLE brain correlate with cognitive impairment. JCI Insight.

[bib33] Mayer C., Nägele F.L., Petersen M., Frey B.M., Hanning U., Pasternak O. (2022). Free-water diffusion MRI detects structural alterations surrounding white matter hyperintensities in the early stage of cerebral small vessel disease. J. Cerebr. Blood Flow Metabol..

[bib34] Mikdashi J.A., Esdaile J.M., Alarcón G.S., Crofford L., Fessler B.J. (2007). Proposed response criteria for neurocognitive impairment in systemic lupus erythematosus clinical trials. Lupus.

[bib35] Nichilatti L.P., Fernandes J.M., Marques C.P. (2020). Physiopathology of pain in systemic erythematosus lupus. Lupus.

[bib36] Nystedt J., Mannfolk P., Jönsen A., Nilsson P., Strandberg T.O., Sundgren P.C. (2019). Functional connectivity changes in core resting state networks are associated with cognitive performance in systemic lupus erythematosus. J. Comp. Neurol..

[bib37] Oh D.H., Kim S.H., Jung S., Sung Y.K., Bang S.Y., Bae S.C. (2011). Precuneus hypoperfusion plays an important role in memory impairment of patients with systemic lupus erythematosus. Lupus.

[bib38] Olazarán J., López-Longo J., Cruz I., Bittini A., Carreño L. (2009). Cognitive dysfunction in systemic lupus erythematosus: prevalence and correlates. Eur. Neurol..

[bib39] Omdal R., Sjöholm H., Koldingsnes W., Sundsfjord J.A., Jacobsen E.A., Husby G. (2005). Fatigue in patients with lupus is not associated with disturbances in cerebral blood flow as detected by SPECT. J. Neurol..

[bib40] Papadaki E., Kavroulakis E., Bertsias G., Fanouriakis A., Karageorgou D., Sidiropoulos P. (2019). Regional cerebral perfusion correlates with anxiety in neuropsychiatric SLE: evidence for a mechanism distinct from depression. Lupus.

[bib41] Perneger T.V. (1998). What's wrong with Bonferroni adjustments. BMJ.

[bib42] Petri M., Orbai A.-M., Alarcón G.S., Gordon C., Merrill J.T., Fortin P.R. (2012). Derivation and validation of the Systemic Lupus International Collaborating Clinics classification criteria for systemic lupus erythematosus. Arthritis Rheum..

[bib43] Qian X., Ji F., Ng K.K., Koh A.J., Loo B.R.Y., Townsend M.C. (2022). Brain white matter extracellular free-water increases are related to reduced neurocognitive function in systemic lupus erythematosus. Rheumatology.

[bib44] Quarles C.C., Gochberg D.F., Gore J.C., Yankeelov T.E. (2009). A theoretical framework to model DSC-MRI data acquired in the presence of contrast agent extravasation. Phys. Med. Biol..

[bib45] Rayes H.A., Tani C., Kwan A., Marzouk S., Colosimo K., Medina-Rosas J. (2018). What is the prevalence of cognitive impairment in lupus and which instruments are used to measure it? A systematic review and meta-analysis. Semin. Arthritis Rheum..

[bib46] Salomonsson T., Rumetshofer T., Jönsen A., Bengtsson A.A., Zervides K.A., Nilsson P. (2023). Abnormal cerebral hemodynamics and blood-brain barrier permeability detected with perfusion MRI in systemic lupus erythematosus patients. Neuroimage Clin.

[bib47] Schulz M., Engelhardt B. (2005). The circumventricular organs participate in the immunopathogenesis of experimental autoimmune encephalomyelitis. Cerebrospinal Fluid Res..

[bib48] Seet D., Allameen N.A., Tay S.H., Cho J., Mak A. (2021). Cognitive dysfunction in systemic lupus erythematosus: immunopathology, clinical manifestations, neuroimaging and management. Rheumatol Ther.

[bib49] Shi Y., Li M., Liu L., Wang Z., Wang Y., Zhao J. (2021). Relationship between disease activity, organ damage and health-related quality of life in patients with systemic lupus erythematosus: a systemic review and meta-analysis. Autoimmun. Rev..

[bib50] Shokouhi M., Davis K.D., Moulin D.E., Morley-Forster P., Nielson W.R., Bureau Y. (2016). Basal ganglia perfusion in fibromyalgia is related to pain disability and disease impact: an arterial spin labeling study. Clin. J. Pain.

[bib51] Stock A.D., Gelb S., Pasternak O., Ben-Zvi A., Putterman C. (2017). The blood brain barrier and neuropsychiatric lupus: new perspectives in light of advances in understanding the neuroimmune interface. Autoimmun. Rev..

[bib52] (1999). The American College of Rheumatology nomenclature and case definitions for neuropsychiatric lupus syndromes. Arthritis Rheum..

[bib53] Trautmann A. (2021). Mechanisms underlying chronic fatigue, a symptom too often overlooked II- from deregulated immunity to neuroinflammation and its consequences. Med. Sci..

[bib54] Unterman A., Nolte J.E.S., Boaz M., Abady M., Shoenfeld Y., Zandman-Goddard G. (2011). Neuropsychiatric syndromes in systemic lupus erythematosus: a meta-analysis. Semin. Arthritis Rheum..

[bib55] Vo A., Volpe B.T., Tang C.C., Schiffer W.K., Kowal C., Huerta P.T. (2014). Regional brain metabolism in a murine systemic lupus erythematosus model. J. Cerebr. Blood Flow Metabol..

[bib56] Wang P.I., Cagnoli P.C., McCune W.J., Schmidt-Wilcke T., Lowe S.E., Graft C.C. (2012). Perfusion-weighted MR imaging in cerebral lupus erythematosus. Acad. Radiol..

[bib58] Wang L., Zheng G., Jia X., Zhang X., Chen Y. (2023). Application study of brain structure and functional magnetic resonance imaging in patients with systemic lupus erythematosus and cognitive dysfunction. Alternative Ther. Health Med..

[bib59] Wasan A.D., Loggia M.L., Chen L.Q., Napadow V., Kong J., Gollub R.L. (2011). Neural correlates of chronic low back pain measured by arterial spin labeling. Anesthesiology.

[bib60] Waterloo K., Omdal R., Sjöholm H., Koldingsnes W., Jacobsen E.A., Sundsfjord J.A. (2001). Neuropsychological dysfunction in systemic lupus erythematosus is not associated with changes in cerebral blood flow. J. Neurol..

[bib61] Wiseman S.J., Bastin M.E., Jardine C.L., Barclay G., Hamilton I.F., Sandeman E. (2016). Cerebral small vessel disease burden is increased in systemic lupus erythematosus. Stroke.

[bib62] Wolfe F., Smythe H.A., Yunus M.B., Bennett R.M., Bombardier C., Goldenberg D.L. (1990). The American College of Rheumatology 1990 criteria for the classification of fibromyalgia. Report of the multicenter criteria committee. Arthritis Rheum..

[bib63] Zhuo Z., Su L., Duan Y., Huang J., Qiu X., Haller S. (2020). Different patterns of cerebral perfusion in SLE patients with and without neuropsychiatric manifestations. Hum. Brain Mapp..

