# Supplementary material for: Brain perfusion and blood-brain barrier permeability in systemic lupus erythematosus patients: Associations with disease activity, cognitive dysfunction, fatigue and pain
Source: Neuroimage Rep. 2024 Dec 31;5(1):100232. doi: 10.1016/j.ynirp.2024.100232 (PMC12172694; doi:10.1016/j.ynirp.2024.100232)
Supplement: Multimedia component 1 [file mmc1.docx]

**SUPPLEMENTARY MATERIAL**

Brain perfusion and blood-brain barrier permeability in systemic lupus erythematosus patients: Associations with disease activity, cognitive dysfunction, fatigue and pain.

Tim Salomonsson ^a^, Kristoffer A Zervides ^b^, Andreas Jönsen ^b^, Malte Knutsson ^a^, Ronnie Wirestam ^c^, Jimmy Lätt ^d^, Anders A Bengtsson ^b^, *Linda Knutsson ^c,e,f^, *Pia C Sundgren ^a,d,g^

a. Department of Clinical Sciences, Diagnostic Radiology, Lund University, Skåne University Hospital, 221 85 Lund, Sweden.

b. Department of Clinical Sciences, Rheumatology, Lund University, Skåne University Hospital, 222 42 Lund, Sweden.

c. Department of Medical Radiation Physics, Lund University, 221 85 Lund, Sweden.

d. Department of Medical Imaging and Physiology, Skåne University Hospital, 221 85 Lund, Sweden.

e. Department of Neurology, Johns Hopkins University School of Medicine, Baltimore, MD 21287, United States.

f. F.M. Kirby Research Center for Functional Brain Imaging, Kennedy Krieger Institute, Baltimore, MD 21205, United States.

g. Lund University Bioimaging Center, Lund University, 221 84 Lund, Sweden.

* Shared last authors.

**Corresponding author**

Pia C Sundgren, MD., PhD.

Professor of Radiology

Head of the Department of Diagnostic Radiology, Clinical Sciences Lund

Co-Director for Lund University BioImaging Center (LBIC), Lund University

Senior consultant in Neuroradiology

Center for Medical Imaging and Physiology

Skåne University Hospital

SE.221 85 Lund, Sweden

Phone +46706241561

e-mail: [pia.sundgren@med.lu.se](mailto:pia.sundgren@med.lu.se)

**Supplementary table 1.** Standard scores and prevalence of mild and moderate cognitive deficits in different domains using CNS-VS in 65 SLE patients, sorted by single test domain, multiple test domain and NCI.

| Cognitive domain | Valid tests | Median ss (range) | Mild deficit (%)^a^ | Moderate deficit (%)^b^ |
| --- | --- | --- | --- | --- |
| Verbal memory | 65 | 99 (39–126) | 14 (21 %) | 7 (11 %) |
| Visual memory | 65 | 96 (56–125) | 13 (20 %) | 8 (13 %) |
| Motor speed | 37 | 94 (77–124) | 5 (8 %) | 2 (3 %) |
| Processing speed | 65 | 101 (66–132) | 11 (17 %) | 4 (6 %) |
| Executive function | 64 | 98 (38–130) | 12 (18 %) | 7 (11 %) |
| Simple attention | 36 | 106 (57–112) | 6 (9 %) | 4 (6 %) |
| Reaction time | 64 | 92 (20–119) | 23 (36 %) | 15 (23 %) |
| Composite memory^c^ | 65 | 97 (47–127) | 15 (23 %) | 8 (13 %) |
| Psychomotor speed^d^ | 65 | 96 (68–129) | 10 (15 %) | 3 (5 %) |
| Cognitive flexibility^e^ | 63 | 98 (35–129) | 11 (17 %) | 7 (11 %) |
| Complex attention^f^ | 62 | 103 (25–120) | 9 (14 %) | 7 (11 %) |
| NCI | 62 | 95 (50–114) | 8 (12 %) | 4 (6 %) |

SLE = Systemic lupus erythematosus, CNS-VS = Central nervous system-Vital Signs, ss = Standard score, NCI = Neurocognition index.

^a^Number and percentage of SLE patients with cognitive deficit in each domain, defined as standard score ≤ 85, corresponding with a deviation ≤ –1 SD of the age-matched expected value.

^b^Number and percentage of SLE patients with cognitive deficit in each domain, defined as standard score ≤ 79, corresponding with a deviation ≤ –1.4 SD of the age-matched expected value.

^c^Combining the performance in the visual and verbal memory domains.

^d^Combining the performance in the motor speed and processing speed domains.

^e^Combining the performance in the executive function and reaction time domains.

^f^Combining the performance in the executive function, simple attention and reaction time domains.

^g^Number of patients with cognitive deficits in a single domain.

^h^Number of patients with cognitive deficits in two or more domains.

**Supplementary table 2.** Correlations between K_2_ and standard scores in the investigated cognitive domains in SLE, presented with partial correlation coefficients and p values. Correlation coefficients ≥ 0.4 or ≤ –0.4 and p values < 0.05 are marked in bold.

|  | | NCI | Motor speed | Simple attention | Visual memory | Verbal memory | Executive function | Processing speed | Cognitive flexibility | Complex attention | Reaction time | Psychomotor speed | Composite memory |
| --- | --- | --- | --- | --- | --- | --- | --- | --- | --- | --- | --- | --- | --- |
| Patients, n | | 56 | 31 | 30 | 59 | 59 | 58 | 59 | 57 | 56 | 58 | 59 | 59 |
| R MTL K_2_ | Correlation | 0.11 | −0.11 | 0.04 | 0.24 | 0.26 | 0.00 | 0.25 | 0.01 | 0.09 | −0.03 | 0.14 | 0.30 |
|  | P value | 0.42 | 0.53 | 0.85 | 0.06 | **0.05** | 1.00 | 0.05 | 0.95 | 0.52 | 0.83 | 0.29 | **0.02** |
| L MTL K_2_ | Correlation | −0.06 | −0.28 | **−0.50** | 0.02 | 0.07 | −0.11 | 0.07 | −0.11 | −0.11 | −0.10 | −0.10 | 0.06 |
|  | P value | 0.66 | 0.12 | **< 0.01** | 0.89 | 0.57 | 0.41 | 0.62 | 0.41 | 0.40 | 0.47 | 0.45 | 0.67 |
| R Ant. insula K_2_ | Correlation | −0.02 | −0.07 | −0.11 | 0.15 | 0.07 | −0.06 | 0.13 | −0.06 | −0.08 | −0.04 | 0.06 | 0.13 |
|  | P value | 0.86 | 0.72 | 0.56 | 0.26 | 0.61 | 0.63 | 0.32 | 0.64 | 0.54 | 0.75 | 0.64 | 0.31 |
| L Ant. insula K_2_ | Correlation | −0.23 | −0.23 | **−0.48** | 0.06 | −0.14 | −0.20 | −0.02 | −0.18 | −0.19 | −0.10 | −0.13 | −0.06 |
|  | P value | 0.09 | 0.21 | **0.01** | 0.65 | 0.28 | 0.13 | 0.89 | 0.19 | 0.15 | 0.45 | 0.32 | 0.63 |
| R Post. insula K_2_ | Correlation | −0.30 | **−0.40** | −0.35 | −0.15 | −0.16 | −0.28 | −0.27 | −0.30 | −0.32 | −0.19 | −0.31 | −0.18 |
|  | P value | **0.02** | **0.02** | 0.05 | 0.25 | 0.21 | **0.03** | **0.03** | **0.02** | **0.02** | 0.15 | **0.02** | 0.17 |
| L Post. insula K_2_ | Correlation | −0.09 | −0.12 | 0.11 | 0.10 | 0.04 | −0.13 | 0.05 | −0.16 | −0.16 | −0.09 | −0.02 | 0.09 |
|  | P value | 0.52 | 0.53 | 0.55 | 0.45 | 0.76 | 0.32 | 0.72 | 0.23 | 0.23 | 0.50 | 0.88 | 0.51 |
| R Hypothalamus K_2_ | Correlation | −0.28 | −0.07 | 0.18 | −0.20 | −0.10 | −0.24 | 0.15 | −0.22 | −0.16 | −0.25 | 0.00 | −0.17 |
|  | P value | **0.03** | 0.71 | 0.34 | 0.13 | 0.47 | 0.06 | 0.26 | 0.10 | 0.25 | 0.06 | 0.97 | 0.19 |
| L Hypothalamus K_2_ | Correlation | −0.22 | 0.02 | −0.30 | −0.08 | −0.16 | −0.17 | 0.12 | −0.14 | −0.06 | −0.28 | 0.00 | −0.15 |
|  | P value | 0.09 | 0.92 | 0.10 | 0.52 | 0.22 | 0.20 | 0.36 | 0.30 | 0.63 | **0.03** | 0.98 | 0.26 |
| R Nc. caudatus K_2_ | Correlation | −0.06 | 0.04 | −0.27 | 0.01 | −0.20 | 0.03 | 0.02 | 0.03 | 0.05 | −0.15 | 0.03 | −0.13 |
|  | P value | 0.67 | 0.82 | 0.15 | 0.96 | 0.12 | 0.80 | 0.90 | 0.83 | 0.70 | 0.27 | 0.80 | 0.32 |
| L Nc. caudatus K_2_ | Correlation | −0.11 | 0.00 | 0.01 | −0.05 | 0.00 | −0.20 | 0.19 | −0.18 | −0.07 | −0.01 | 0.10 | −0.03 |
|  | P value | 0.40 | 0.99 | 0.97 | 0.72 | 0.99 | 0.14 | 0.15 | 0.18 | 0.58 | 0.92 | 0.43 | 0.80 |
| R Putamen K_2_ | Correlation | **−0.50** | −0.12 | −0.26 | −0.18 | −0.25 | **−0.44** | −0.04 | **−0.42** | **−0.41** | −0.37 | −0.10 | −0.26 |
|  | P value | **< 0.01** | 0.53 | 0.15 | 0.16 | 0.05 | **< 0.01** | 0.77 | **< 0.01** | **< 0.01** | **< 0.01** | 0.43 | **0.04** |
| L Putamen K_2_ | Correlation | −0.35 | −0.09 | −0.17 | −0.16 | −0.20 | −0.29 | 0.09 | −0.31 | −0.38 | −0.06 | −0.07 | −0.21 |
|  | P value | **0.01** | 0.64 | 0.36 | 0.21 | 0.13 | **0.03** | 0.50 | **0.02** | **< 0.01** | 0.63 | 0.62 | 0.10 |
| R Thalamus K_2_ | Correlation | −0.10 | 0.11 | **−0.46** | −0.25 | −0.23 | 0.03 | −0.03 | 0.05 | 0.03 | −0.20 | 0.05 | −0.29 |
|  | P value | 0.45 | 0.54 | **0.01** | 0.06 | 0.08 | 0.81 | 0.81 | 0.69 | 0.85 | 0.14 | 0.73 | **0.03** |
| L Thalamus K_2_ | Correlation | −0.30 | −0.05 | **−0.52** | −0.15 | −0.28 | −0.21 | −0.22 | −0.19 | −0.17 | −0.25 | −0.14 | −0.26 |
|  | P value | **0.03** | 0.78 | **< 0.01** | 0.24 | **0.03** | 0.11 | 0.09 | 0.16 | 0.20 | 0.05 | 0.29 | **0.04** |
| R Frontal WM K_2_ | Correlation | 0.01 | 0.20 | −0.02 | −0.03 | −0.07 | 0.04 | 0.00 | 0.03 | 0.01 | 0.13 | 0.00 | −0.06 |
|  | P value | 0.94 | 0.27 | 0.92 | 0.83 | 0.60 | 0.79 | 0.98 | 0.80 | 0.96 | 0.34 | 0.98 | 0.65 |
| L Frontal WM K_2_ | Correlation | 0.02 | 0.13 | 0.35 | −0.11 | 0.06 | 0.09 | −0.10 | 0.03 | −0.09 | 0.23 | −0.09 | −0.02 |
|  | P value | 0.88 | 0.47 | 0.05 | 0.41 | 0.68 | 0.52 | 0.46 | 0.80 | 0.51 | 0.08 | 0.50 | 0.89 |
| Ant. corpus callosum K_2_ | Correlation | −0.01 | 0.16 | 0.17 | −0.27 | 0.08 | 0.04 | −0.21 | 0.05 | 0.03 | 0.06 | −0.01 | −0.10 |
|  | P value | 0.93 | 0.39 | 0.37 | **0.04** | 0.55 | 0.75 | 0.10 | 0.72 | 0.84 | 0.64 | 0.95 | 0.45 |
| Post. corpus callosum K_2_ | Correlation | 0.01 | −0.06 | −0.12 | −0.08 | 0.08 | 0.07 | 0.09 | 0.07 | 0.01 | −0.19 | 0.05 | 0.02 |
|  | P value | 0.93 | 0.74 | 0.53 | 0.57 | 0.55 | 0.62 | 0.51 | 0.63 | 0.96 | 0.15 | 0.70 | 0.88 |
| R DL PFC K_2_ | Correlation | −0.07 | −0.08 | **−0.62** | 0.00 | −0.03 | 0.00 | −0.05 | 0.01 | −0.05 | −0.24 | −0.06 | −0.02 |
|  | P value | 0.61 | 0.65 | **< 0.01** | 0.99 | 0.85 | 0.99 | 0.71 | 0.94 | 0.70 | 0.07 | 0.62 | 0.90 |
| L DL PFC K_2_ | Correlation | −0.09 | 0.01 | **−0.53** | −0.03 | −0.05 | −0.04 | −0.14 | −0.01 | 0.05 | −0.27 | −0.09 | −0.05 |
|  | P value | 0.52 | 0.97 | **< 0.01** | 0.82 | 0.71 | 0.76 | 0.28 | 0.93 | 0.69 | **0.04** | 0.48 | 0.71 |
| R VM PFC K_2_ | Correlation | −0.22 | −0.29 | −0.31 | −0.20 | −0.04 | −0.21 | −0.16 | −0.19 | −0.16 | −0.32 | −0.27 | −0.14 |
|  | P value | 0.10 | 0.10 | 0.09 | 0.12 | 0.75 | 0.11 | 0.21 | 0.15 | 0.22 | **0.01** | **0.04** | 0.27 |
| L VM PFC K_2_ | Correlation | −0.28 | −0.29 | −0.20 | −0.17 | −0.16 | −0.25 | −0.14 | −0.25 | −0.29 | −0.23 | −0.21 | −0.20 |
|  | P value | **0.04** | 0.11 | 0.29 | 0.18 | 0.23 | 0.06 | 0.28 | 0.06 | **0.03** | 0.08 | 0.10 | 0.13 |
| R Ant. cingulate cortex K_2_ | Correlation | −0.14 | −0.05 | −0.19 | −0.09 | −0.08 | −0.01 | 0.08 | 0.02 | 0.04 | −0.27 | −0.05 | −0.11 |
|  | P value | 0.30 | 0.80 | 0.30 | 0.49 | 0.54 | 0.97 | 0.53 | 0.89 | 0.75 | **0.04** | 0.69 | 0.42 |
| L Ant. cingulate cortex K_2_ | Correlation | −0.03 | −0.05 | **−0.51** | −0.09 | 0.00 | −0.03 | −0.11 | −0.02 | 0.05 | −0.15 | −0.10 | −0.05 |
|  | P value | 0.82 | 0.80 | **< 0.01** | 0.49 | 1.00 | 0.80 | 0.39 | 0.89 | 0.70 | 0.24 | 0.46 | 0.73 |
| R Post. cingulate cortex K_2_ | Correlation | −0.25 | −0.26 | **−0.45** | −0.33 | −0.15 | −0.15 | −0.11 | −0.14 | −0.26 | −0.27 | −0.10 | −0.28 |
|  | P value | 0.06 | 0.14 | **0.01** | **0.01** | 0.27 | 0.27 | 0.39 | 0.31 | 0.05 | **0.04** | 0.44 | **0.03** |
| L Post. cingulate cortex K_2_ | Correlation | −0.35 | −0.15 | **−0.66** | −0.16 | −0.29 | −0.28 | −0.16 | −0.25 | −0.26 | −0.33 | −0.08 | −0.29 |
|  | P value | **0.01** | 0.42 | **< 0.01** | 0.21 | **0.02** | **0.03** | 0.22 | 0.06 | **0.05** | **0.01** | 0.55 | **0.03** |
| R SPL K_2_ | Correlation | −0.22 | −0.05 | **−0.60** | −0.05 | −0.26 | −0.18 | 0.10 | −0.14 | −0.17 | −0.24 | 0.03 | −0.20 |
|  | P value | 0.09 | 0.77 | **< 0.01** | 0.71 | **0.04** | 0.18 | 0.46 | 0.29 | 0.20 | 0.07 | 0.81 | 0.13 |
| L SPL K_2_ | Correlation | −0.15 | −0.02 | −0.34 | −0.02 | −0.17 | −0.18 | −0.09 | −0.15 | −0.05 | −0.25 | −0.03 | −0.12 |
|  | P value | 0.27 | 0.90 | 0.06 | 0.87 | 0.20 | 0.18 | 0.50 | 0.27 | 0.68 | 0.06 | 0.81 | 0.34 |
| Average K_2_ | Correlation | −0.38 | −0.21 | **−0.61** | −0.22 | −0.20 | −0.30 | −0.04 | −0.28 | −0.27 | **−0.42** | −0.13 | −0.25 |
|  | P value | **< 0.01** | 0.24 | **< 0.01** | 0.10 | 0.14 | **0.02** | 0.76 | **0.04** | **0.04** | **< 0.01** | 0.32 | 0.06 |

K_2_ = Blood-brain barrier leakage parameter, SLE = Systemic lupus erythematosus, L = Left, R = Right, Nc. = Nucleus, Ant. = Anterior, Post. = Posterior, WM = White matter, MTL = Medial temporal lobe, PFC = Prefrontal cortex, DL = Dorsolateral, VM = Ventromedial, SPL = Superior parietal lobule.

**Supplementary table 3.** Normalized perfusion-based values (CBF, CBV and MTT) and absolute values of the leakage parameter K_2_, in the regions of interest that displayed significant differences between SLE patients with and without fibromyalgia.

| Region of interest | Control | Symptom | P value |
| --- | --- | --- | --- |
| Fibromyalgia | | | |
| R MTL CBF | 1.62 | 2.05 | 0.02 |
| R MTL CBV | 1.51 | 1.93 | 0.03 |
| L MTL CBF | 1.68 | 2.10 | 0.02 |
| L MTL CBV | 1.55 | 1.90 | 0.03 |
| R Ant. insula CBV | 1.61 | 1.97 | 0.03 |
| R Post. insula CBF | 1.87 | 2.39 | 0.01 |
| R Post. insula CBV | 1.56 | 2.03 | < 0.01 |
| R Hypothalamus CBF | 2.20 | 2.59 | 0.04 |
| R Hypothalamus CBV | 1.97 | 2.34 | 0.04 |
| L Hypothalamus CBF | 2.21 | 2.65 | 0.03 |
| R Nc. caudatus CBF | 2.83 | 3.35 | 0.05 |
| L Nc. caudatus CBF | 2.86 | 3.56 | 0.02 |
| L Nc. caudatus CBV | 2.27 | 2.73 | 0.03 |
| R Putamen CBF | 2.54 | 3.40 | < 0.01 |
| R Putamen CBV | 1.90 | 2.36 | 0.01 |
| L Putamen CBF | 2.59 | 3.33 | 0.01 |
| L Putamen CBV | 1.90 | 2.32 | 0.02 |
| R Thalamus CBF | 1.97 | 2.66 | < 0.01 |
| R Thalamus MTT | 0.93 | 0.82 | 0.01 |
| L Thalamus CBF | 2.05 | 2.64 | 0.01 |
| L Thalamus MTT | 0.94 | 0.85 | 0.02 |
| R Frontal WM CBF | 0.81 | 0.98 | 0.04 |
| R Frontal WM CBV | 0.84 | 1.03 | 0.03 |
| L Frontal WM CBF | 0.81 | 1.05 | < 0.01 |
| L Frontal WM CBV | 0.85 | 1.08 | < 0.01 |
| Ant. corpus callosum CBF | 1.26 | 1.60 | 0.02 |
| R DL PFC CBF | 2.14 | 2.55 | 0.05 |
| R Post. cingulate cortex CBF | 2.34 | 2.88 | 0.04 |
| L Post. cingulate cortex CBF | 2.43 | 3.01 | 0.05 |
| R SPL CBF | 1.61 | 2.09 | < 0.01 |
| R SPL CBV | 1.51 | 1.89 | 0.01 |
| L SPL CBF | 1.64 | 2.19 | < 0.01 |
| L SPL CBV | 1.55 | 2.01 | < 0.01 |
| Average CBF | 2.06 | 2.50 | 0.01 |
| Average CBV | 1.77 | 2.08 | 0.02 |

SLE = Systemic lupus erythematosus, L = Left, R = Right, Nc. = Nucleus, Ant. = Anterior, Post. = Posterior, WM = White matter, MTL = Medial temporal lobe, PFC = Prefrontal cortex, DL = Dorsolateral, VM = Ventromedial, SPL = Superior parietal lobule, CBF = Cerebral blood flow, CBV = Cerebral blood volume, MTT = Mean transit time, CD = Cognitive dysfunction, ADD = Any domain deficit, MDD = Multiple domain deficit.
